# Concealed antiaromaticity

**DOI:** 10.12688/openreseurope.19690.1

**Published:** 2025-03-18

**Authors:** Florian Glöcklhofer

**Affiliations:** 1 Institute of Applied Synthetic Chemistry, TU Wien, Vienna, 1060, Austria; 2 Department of Chemistry, Imperial College London, London, England, W12 0BZ, UK; 3 Centre for Processable Electronics, Imperial College London, London, England, W12 0BZ, UK

**Keywords:** molecular design, conjugated compounds, small molecules, macrocycles, aromaticity, antiaromaticity, concealed antiaromaticity, excited-state aromaticity, stacked-ring aromaticity, redox reactions, photoexictation, intermolecular interactions

## Abstract

The literature reports numerous molecules claimed to be antiaromatic because of a formal 4 *n* π-electron system. However, this neglects the actual local aromaticity of the molecules, which often feature multiple subunits with [4 *n*+2] π-electrons besides the formal 4 *n* π-electron system. This has led to considerable criticism from those who believe that the term antiaromatic should not be used for any molecule with a formal 4 *n* π-electron system but should be reserved for truly antiaromatic molecules. To reconcile the different viewpoints, the concept of concealed antiaromaticity is introduced here. Concealed antiaromaticity acknowledges that many molecules claimed to be antiaromatic are not truly antiaromatic, but they can exhibit behaviour under certain conditions that would normally be expected for antiaromatic molecules. Three types of concealed antiaromaticity are distinguished based on the conditions under which the molecules can behave like antiaromatic molecules: concealed antiaromaticity revealable in redox reactions (Type I-CA), upon photoexcitation (Type II-CA), and in intermolecular interactions (Type III-CA). The concept of concealed antiaromaticity will enable the rational design of molecules that show the desirable properties of antiaromatic molecules under the different conditions, with applications from organic electronics to photoresponsive materials, while avoiding the low stability of truly antiaromatic molecules.

Monocyclic molecules with a delocalized π-electron system of [4 *n*+2] π-electrons, where *n* is a non-negative integer, are aromatic according to Hückel’s rule, whereas with 4 *n* π-electrons they are antiaromatic ^1– 4^. These rules of ground-state aromaticity are reversed in the lowest ππ* singlet and triplet excited states, where molecules with a cyclic delocalized system of 4 *n* π-electrons are expected to be aromatic and with [4 *n*+2] π-electrons to be antiaromatic according to what is known as Baird’s rule ^5– 10^. In all cases, the π-electron system has to be planar or close to planar for the π-electron delocalization to occur. While the π-electron delocalization in aromatic molecules provides for enhanced thermodynamic stability compared to their acyclic structural analogues, antiaromatic molecules are less stable than their acyclic structural analogues ^11^. The lower stability of antiaromatic molecules can be rationalized by their two degenerate non-bonding singly occupied molecular orbitals (SOMOs) that are higher in energy than the highest occupied molecular orbital (HOMO) of the corresponding localized structure. In contrast, aromatic molecules normally feature a closed-shell structure with two degenerate bonding HOMOs that are lower in energy. As a consequence, antiaromatic molecules try to escape antiaromaticity in different ways ^12^, often by adopting a more stable non-planar conformation with localized π-electrons (and a closed-shell structure) or by undergoing reactions. For example, cyclooctatetraene adopts a boat-like non-planar conformation to escape antiaromaticity ( Figure 1a) ^13^, whereas cyclobutadiene is known to dimerize ( Figure 1b) ^14^.

**Figure 1.**
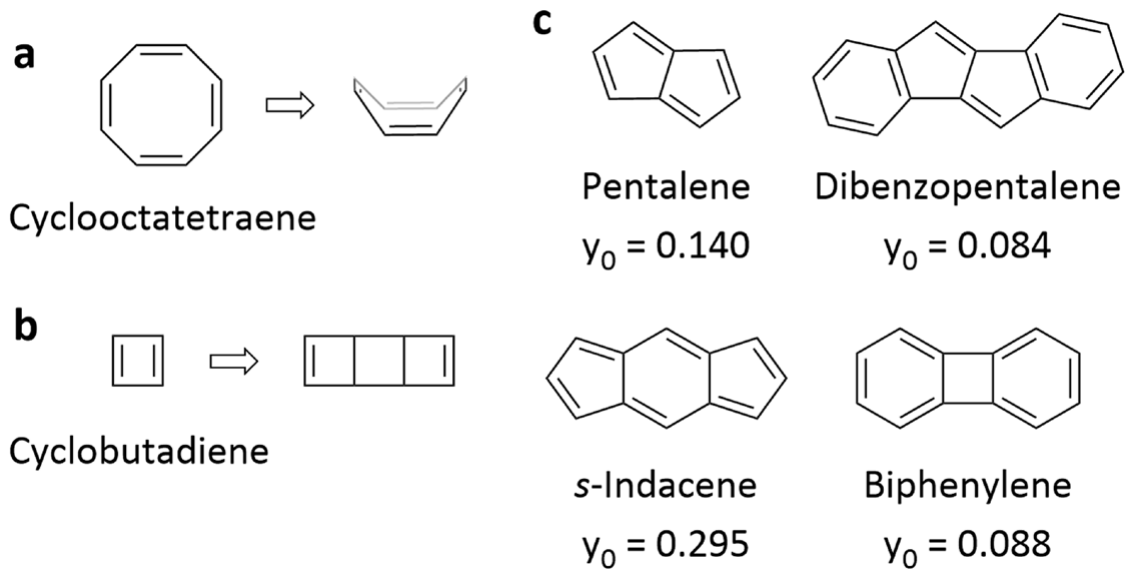
Antiaromaticity escape and diradical character. **a**, **b**, Cyclooctatetraene adopting a non-planar conformation ( **a**) and cyclobutadiene undergoing dimerization ( **b**) to escape antiaromaticity. **c**, Molecular structure and diradical character *y _0_* of pentalene, dibenzopentalene, *s*-indacene, and biphenylene. The diradical character values are from reference 15.

Besides monocyclic antiaromatic molecules with two degenerate SOMOs and a diradical character *y*_0_ of 1 (referred to as truly antiaromatic molecules in this article), there are molecules with a formal 4 *n* π-electron system but reduced diradical character (between 0 and 1) ^15^. These molecules – including pentalene, dibenzopentalene, *s*-indacene, and biphenylene ( Figure 1c) and their derivatives as well as different cyclooctatetraene derivatives – are often also referred to as antiaromatic ^16– 24^, even when the diradical character is low. In contrast to truly antiaromatic molecules, which may only be isolated under cryogenic conditions, these polycyclic molecules are more stable ^12^, with dibenzopentalene and biphenylene being readily isolable. However, the classification of such molecules as antiaromatic has been criticized, particularly for polycyclic molecules that feature both formal 4 *n* and [4 *n*+2] π-electron systems (such as dibenzopentalene and biphenylene, Figure 1c right). At the centre of the controversy is the question of whether any molecule with a formal 4 *n* π-electron system that exhibits some degree of paratropic current (e.g. in ACID plots or according to slightly shifted NMR signals) can be classified as antiaromatic – a viewpoint often held by experimental organic chemists – or whether the term should be reserved for molecules with a 4 *n* π-electron system that fulfil not only the magnetic criterion but also the structural, energetic, and electronic criteria of (anti)aromaticity ^25– 28^. While the latter view follows a rather strict holistic approach and is in better agreement with the definition of (anti)aromaticity ^11^, the former is less restrictive and often based on the observation that the molecules in question behave like antiaromatic molecules under certain conditions, which explains why this viewpoint is often held by experimental chemists. In an approach that generalized Hückel’s rule to polycyclic molecules, such molecules were previously classified as intermediate, showing partially aromatic character ^29^, but this classification does not provide information about the properties and behaviour of the molecules. Other terms that have been suggested in informal discussions, including attenuated antiaromaticity, pseudo-antiaromaticity, or concealed 4 *n* π-electron cycles, are limited in scope and not suitable for the development of a more general framework.

To reconcile the different viewpoints and to enable a rational design of molecules with the observed behaviour, the concept of ‘concealed antiaromaticity’ is introduced here, which provides a more general framework for understanding and explaining the properties that arise from certain structural motifs. The concept accepts that many of the molecules claimed to be antiaromatic due to the presence of a formal 4 *n* π-electron system are not truly antiaromatic. However, at the same time, it acknowledges that this does not prevent those molecules from exhibiting behaviour under certain conditions that would normally be expected for antiaromatic molecules. Quite the contrary, molecules with concealed antiaromaticity allow the desirable properties of antiaromatic molecules under the different conditions to be used without having to deal with their undesirable properties, namely the low stability of truly antiaromatic molecules.

## Structural motifs of concealed antiaromaticity

**Figure 2.**
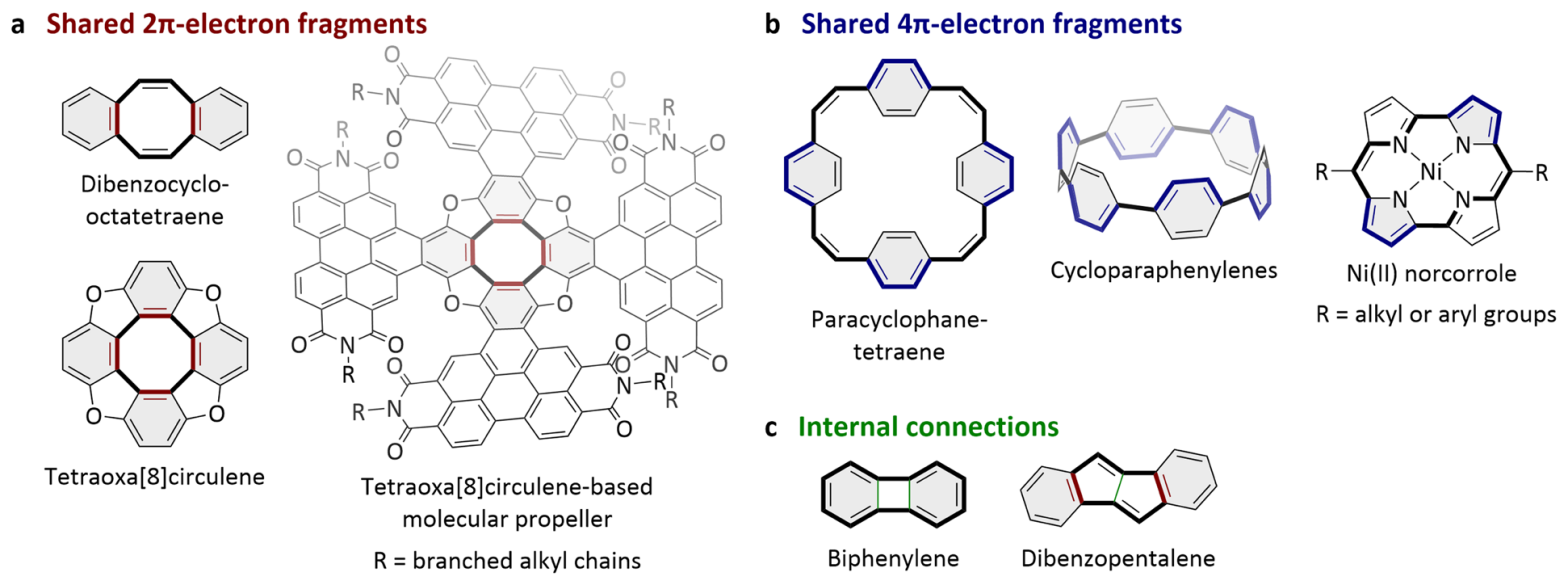
Structural motifs of concealed antiaromaticity. **a**– **c**, Molecules with concealed antiaromaticity, featuring a formal 4 *n* π-electron system (bold bonds) as well as different structural motifs that conceal the antiaromaticity: Locally aromatic subunits (grey shadings) sharing 2 π-electrons (red; **a**) or 4 π-electrons (blue; **b**) with the formal 4 *n* π-electron system or internal connections of the formal 4 *n* π-electron system (green; **c**).

Molecules with concealed antiaromaticity feature a formal 4 *n* π-electron system ( Figure 2, bold bonds), but additional structural units and/or conformational changes prevent this 4 *n* π-electron system from turning the molecules antiaromatic. In other words, the additional structural units and the conformational changes conceal the antiaromaticity, sometimes even resulting in molecules that may also be described as locally aromatic or nonaromatic. Based on molecules described in the literature, three common structural motifs to conceal the antiaromaticity can be identified:

1. **Locally aromatic units sharing 2 π-electrons with the 4** *n* **π-electron system**: Numerous molecules that conceal their antiaromaticity in this way have been reported, ranging from comparatively small molecules like dibenzocyclooctatetraene and tetraoxa[8]circulene to large molecular propellers ( Figure 2a) ^21, 30, 31^. Formulated as a molecular design strategy starting from 4 *n* annulenes, this can be described as *ortho*-fusing aromatic units with the 4 *n* π-electron system.
2. **Locally aromatic units sharing 4 π-electrons with the 4** *n* **π-electron system**: Conjugated macrocycles often conceal their antiaromaticity in this way, including paracyclophanetetraene and cycloparaphenylenes ( Figure 2b) ^32– 34^. Formulated as a molecular design strategy, this can be described as creating locally aromatic units along the 4 *n* π-electron system by introducing 2π-electron bridges.

In both cases, fragments of the 4 *n* π-electron system are integrated into locally aromatic subunits with [4 *n*+2] π-electrons ( Figure 2, grey shadings), but the integration of 4 π-electrons is considered to have a more pronounced effect on the antiaromaticity than the integration of 2 π-electrons. Both structural motifs can also be present in the same molecule ^35, 36^. Aromatic units may also be fused with more than one 4 *n* π-electron system ^37^. In addition to these units, the molecules are often able to further conceal the antiaromaticity by conformational changes, unless structural restrictions prevent the formal 4 *n* π-electron system from adopting a non-planar conformation.

3. **Internal connections of the 4** *n* **π-electron system**: Covalent bonds that connect atoms of the 4 *n* π-electron system directly, so without an additional structural unit, can conceal the antiaromaticity / reduce the diradical character ^15^. This is particularly effective if it creates locally aromatic units (in which all π-electrons of these locally aromatic units also form part of the formal 4 *n* π-electron system), as in biphenylene ( Figure 2c left). However, internal connections can considerably reduce the ability of the molecules to further conceal the antiaromaticity by conformational changes. Therefore, this structural motif is often found in combination with other structural motifs (especially when the internal connection does not create locally aromatic units), as in dibenzopentalene ( Figure 2c right). As a molecular design strategy, connecting the 4 *n* π-electron system internally offers less flexibility, as no additional atoms are introduced.

Substituents at the 4 *n* π-electron system or heteroatoms in the 4 *n* π-electron system can also have an impact on the level of concealment but are normally only found in combination with one or more of the structural motifs outlined above. For example, Ni(II) norcorrole features two nitrogen atoms in the formal 4 *n* π-electron system, two locally aromatic subunits that share 4 π-electrons with the formal 4 *n* π-electron system, as well as two vinylene substituents that bridge the π-electron system but do not create locally aromatic units ( Figure 2b right). The substitution of carbon atoms in the 4 *n* π-electron system with nitrogen atoms, as in Ni(II) norcorroles and as studied computationally for pentalene ^38^, is considered to add to the level of concealment. Other structural modifications such as the coordination to a metal atom or ion, as in cyclobutadiene–metal complexes or in Ni(II) norcorroles, may also conceal the antiaromaticity or contribute to the concealment.

## Three types of concealed antiaromaticity

Concealing the antiaromaticity increases the stability of the molecules during synthesis, purification, and processing while retaining desirable properties of antiaromatic molecules (e.g. excellent stability in the doubly charged states), but which properties are retained depends on how strongly the antiaromaticity is concealed. The integration of fragments of the 4 *n* π-electron system into locally aromatic subunits is considered to lead to a tug-of-war between the local aromaticity of the subunits and the global antiaromaticity of the 4 *n* π-electron system, which can be influenced by (i) the choice of subunits and the strength of their local aromaticity, (ii) the number of aromatic subunits in relation to the number of π-electrons in the 4 *n* π-electron system, and (iii) the ability or disability of the 4 *n* π-electron system to undergo conformational changes. Depending on these factors, different degrees of concealment can be achieved. The antiaromaticity can be strongly concealed like in cycloparaphenylenes ( Figure 2b centre), which feature a high number of strongly aromatic subunits in relation to the number of π-electrons in the formal 4 *n* π-electron system, or very weakly concealed like in Ni(II) norcorroles ( Figure 2b right), which cannot undergo conformational changes due to structural restrictions and only features two locally aromatic subunits in the formal 16 π-electron system, resulting in a diradical character *y*_0_ of 0.127 ^39^, similar to the diradical character of pentalene. If the antiaromaticity is strongly concealed, the molecules may also be seen as locally aromatic (or nonaromatic in cases like cyclooctatetraene), but also for these molecules the term concealed antiaromaticity can provide useful guidance to understand the experimental properties, as these molecules can still behave like antiaromatic molecules under certain conditions. The degree of concealment determines the conditions under which the molecules can behave like antiaromatic molecules, so the conditions under which they can reveal their concealed antiaromaticity. Based on these conditions, three types of concealed antiaromaticity are distinguished here:

1. **Type I-CA**: Concealed antiaromaticity revealable in redox reactions
2. **Type II-CA**: Concealed antiaromaticity revealable upon photoexcitation
3. **Type III-CA**: Concealed antiaromaticity revealable in intermolecular interactions

**Figure 3.**
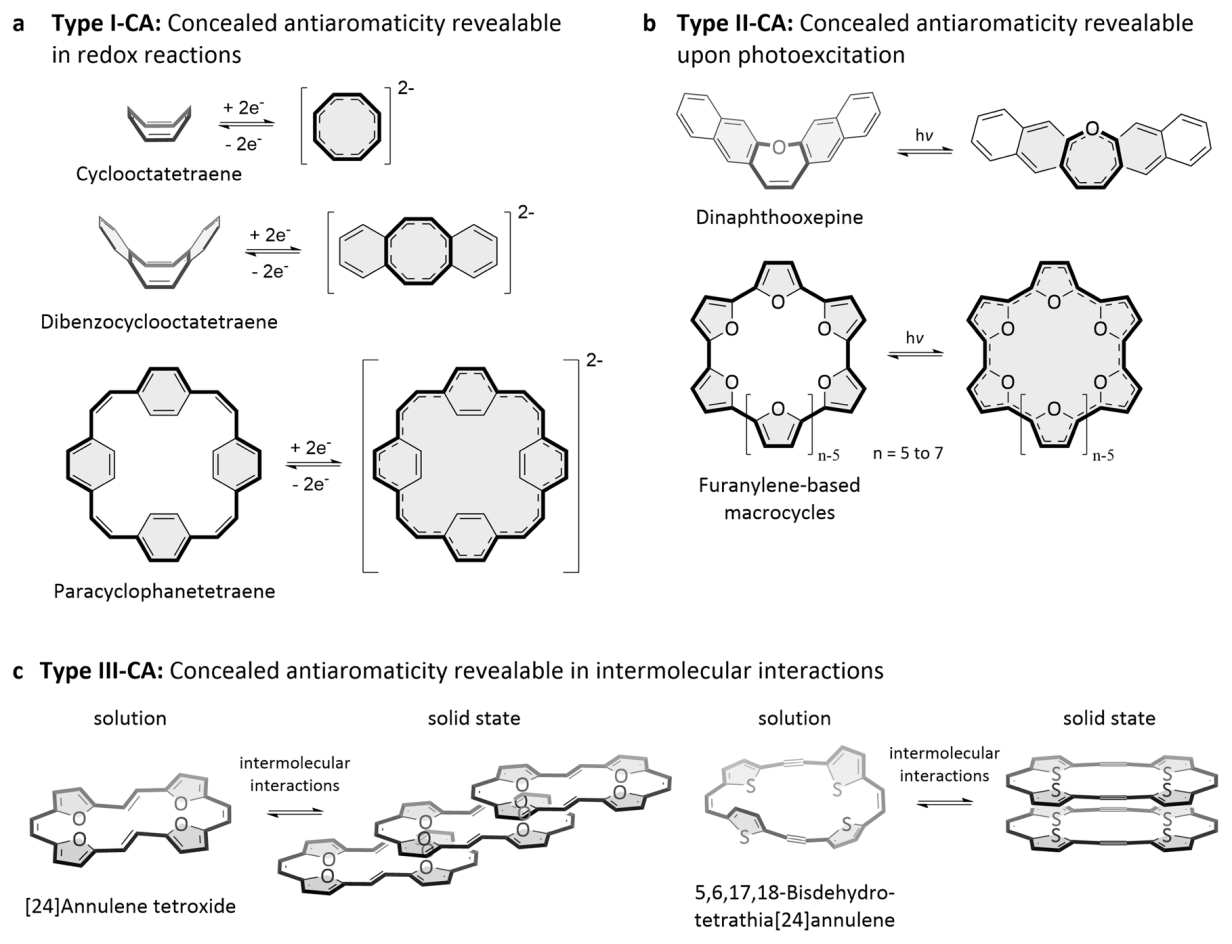
Three types of concealed antiaromaticity. **a**– **c**, Examples of molecules with concealed antiaromaticity revealable in redox reactions (Type I-CA; **a**), upon photoexcitation (Type II-CA; **b**), and in intermolecular interactions (Type III-CA; **c**). **Type I-CA**: Cyclooctatetraene and dibenzocyclooctatetraene planarize and become aromatic upon twofold reduction. Paracyclophanetetraene cannot fully planarize but can still become globally aromatic in redox reactions. **Type II-CA**: Dinaphthooxepine and furanylene-based macrocycles planarize and become Baird aromatic upon photoexcitation. **Type III-CA**: [24]Annulene tetroxide and 5,6,17,18-bisdehydrotetrathia[24]annulene planarize and show chain-like/dimer stacking in the solid state.

The different types of concealed antiaromaticity are not mutually exclusive. Molecules with Type I-CA often also feature Type II-CA and vice versa. Molecules with Type III-CA will normally also feature Type I-CA and Type II-CA. The three types of concealed antiaromaticity are discussed in more detail below, along with a selection of examples identified in the literature (see also Figure 3).

**Concealed antiaromaticity revealable in redox reactions (Type I-CA):** Molecules with Type I-CA conceal their antiaromaticity in the neutral state but behave like antiaromatic molecules when reduced or oxidized to the doubly charged states, where – just like truly antiaromatic molecules – the molecules become globally aromatic. This is because the formal 4 *n* π-electron system transitions to a [4 *n*+2] π-electron system upon twofold reduction or oxidation. For molecules that can conceal their antiaromaticity by conformational changes, the reduction/oxidation often goes along with a planarization of the involved π-electron system and a loss of the conformational flexibility. The planarization enhances the π-electron delocalization in the system, which is energetically favourable in the doubly charged, aromatic states. However, the molecules can also become globally aromatic if full planarization is not possible for structural reasons, as other structural changes that result in enhanced π-electron delocalization and reduced bond-length alternation can still be possible.

Type I-CA is the most frequently found type of concealed antiaromaticity in the literature. There are many reports of molecules that become globally aromatic upon twofold reduction or oxidation without being truly antiaromatic in the neutral state and the examples provided here should not be seen as a complete list of molecules exhibiting such behaviour.

As structurally simple examples, both cyclooctatetraene and dibenzocyclooctatetraene planarize and become aromatic upon twofold reduction ( Figure 3a) ^30, 40, 41^. Similarly, dibenzopentalene ( Figure 2c) and its derivatives were shown to become globally aromatic upon reduction to the dianion or oxidation to the dication ^42, 43^. Tetraoxa[8]circulene and the large molecular propeller based on this structure ( Figure 2a) also switch to a globally aromatic state upon reduction ^21^. Similarly, a double aza[7]helicene with a central 8 π-electron unit was shown to become aromatic upon twofold oxidation ^44^. Moving on to larger formal 4 *n* π-electron systems, paracyclophanetetraene ( Figure 3a) and its derivatives were shown to become globally aromatic upon two-electron reduction or oxidation ^45– 51^, although they cannot fully planarize for steric reasons. For paracyclophanetetraene, switching between a locally aromatic neutral state (with strongly concealed antiaromaticity) and a globally aromatic charged state was introduced as a platform for obtaining organic battery electrode materials ^32^; even when integrated into conjugated polymer backbones, this macrocycle retains its ability to become globally aromatic upon reduction ^52^. Ni(II) norcorrole ( Figure 2c) also transitions to a globally aromatic state upon twofold reduction or oxidation, and the effect was used to obtain a bipolar battery electrode material ^53^. However, as the antiaromaticity is only weakly concealed in Ni(II) norcorroles (as discussed above), mesityl substituents were attached to increase the stability by steric protection. Larger macrocycles, such as cycloparaphenylenes ( Figure 2b) of different size, can also reveal their concealed antiaromaticity in redox reactions ^33, 34, 54– 56^. In these macrocycles, each phenylene subunit contributes 4 π-electrons to the formal macrocyclic 4 *n* π-electron system, meaning that all cycloparaphenylenes that were studied featured Type I-CA (independent of the number of subunits). In contrast, each of the repeat units of furanylene-acetylene macrocycles contributes 6 π-electrons to the formal macrocyclic π-electron system, resulting in alternation between formal 4 *n* and [4 *n*+2] π-electron systems with increasing ring size ^57^. Those with a formal 4 *n* π-electron system were more readily oxidized in cyclic voltammetry measurements, indicating Type I-CA. For the neutral macrocycles, the authors compared the properties to those of the corresponding phenylene-acetylene macrocycles, which showed that the antiaromaticity is more strongly concealed with phenylene than with the furanylene subunits. Other examples of macrocycles with Type I-CA include oligofuran and alternating furan-thiophene macrocycles ^58– 60^, expanded carbaisophlorinoids ^61^, heteroatom-substituted porphyrinoids of different size ^62^, and porphyrin-based nanorings ^63, 64^.

**Concealed antiaromaticity revealable upon photoexcitation (Type II-CA):** Molecules with Type II-CA conceal their antiaromaticity in the ground state but behave like antiaromatic molecules when photoexcited to the excited state, where the molecules become Baird aromatic, a behaviour normally ascribed to antiaromatic molecules. As the redox reactions of molecules with Type I-CA, the photoexcitation often goes along with a planarization of the involved π-electron system, but the molecules can also become Baird aromatic if full planarization is not possible for structural reasons.

Many molecules that feature Type I-CA also feature Type II-CA, as shown for cyclooctatetraene, biphenylene, pentalene ( Figure 1) and their derivatives ^65– 70^. Studies on π-expanded oxepines ( Figure 3b) and other 8π-electron heterocycles with locally aromatic units fused with the formal 8π-electron system show that these molecules can also feature Type II-CA ^71– 75^. Moving on to conjugated macrocycles, paracyclophanetetraene ( Figure 3a) and its derivatives also feature Type II-CA according to computations and experimental indications ^47– 50^. The results of computational studies of cycloparaphenylenes ( Figure 2b) of different size and their furanylene analogues ( Figure 3b) show that – with both types of subunits – these macrocycles can feature Type II-CA ^76^. The Baird aromaticity was more pronounced in the macrocycles with furanylene units, in which the antiaromaticity is less concealed due to the weaker local aromaticity of the subunits. For the macrocycles with furanylene-acetylene repeat units (with alternating formal 4 *n* and [4 *n*+2] π-electron systems) discussed in the section on Type I-CA, the pronounced differences in the emission properties depending on the ring size indicate Type II-CA for those macrocycles with a formal 4 *n* π-electron system ^57^. In a host-guest complex of a fullerene and a macrocycle composed of benzene-fused pyrrolo[3,2-b]pyrrole units with Type II-CA ^77^, Baird aromaticity may play a role in rendering the locally excited state at the macrocycle the lowest excited state of the complex. The photoresponsive behaviour of a borole fused with aromatic units may also be explained by Type II-CA ^78^.

**Concealed antiaromaticity revealable in intermolecular interactions (Type III-CA):** Molecules with Type III-CA conceal their antiaromaticity as an individual molecule (in solution) but behave like antiaromatic molecules in the solid state, where energetically favourable intermolecular interactions of the 4 *n* π-electron systems can occur. These interactions can lead to intermolecular π-electron delocalization and stacked-ring aromaticity ^12, 79– 81^, which are highly interesting features for organic electronic materials. In contrast to truly antiaromatic molecules, no covalent bonds between the molecules are formed as a result of the interactions. Unfortunately, however, Type III-CA is the most difficult type of concealed antiaromaticity to achieve, as the antiaromaticity should only be weakly concealed for the molecules to behave like antiaromatic molecules in intermolecular interactions, meaning that the number of aromatic subunits in relation to the number of π-electrons in the 4 *n* π-electron system should be low and no strongly aromatic subunits should be present. This requirement needs to be carefully balanced with the stability requirements. Furthermore, full planarity of the 4 *n* π-electron system in the solid state is important for Type III-CA, as this enables the close, energetically favourable intermolecular interactions of the π-electron systems.

Ni(II) norcorrole derivatives ( Figure 2b) can fulfil these requirements and, indeed, were shown to feature stacked-ring aromaticity when stacked as dimers in micellar capsules or connected via linkers ^79– 81^. The molecular orbital interactions in these systems may be compared to the SOMO-SOMO interactions in double pancake-bonded dimers ^82^. Besides these dimer interactions, also other types of highly interesting solid-state behaviour has been reported for norcorrole derivatives, such as chain-like stacking ^83^, which resembles the staggered, chain-like stacking of organic biradicals that maximize SOMO-SOMO interactions by this type of stacking ^84, 85^, triple-decker stacking ^86^, and one-dimensional supramolecular assembly ^87^. However, as discussed above, bulky substituents can be required to sufficiently increase the stability of Ni(II) norcorrole derivatives for applications, but these hinder the close intermolecular interaction required for Type III-CA.

**Figure 4.**
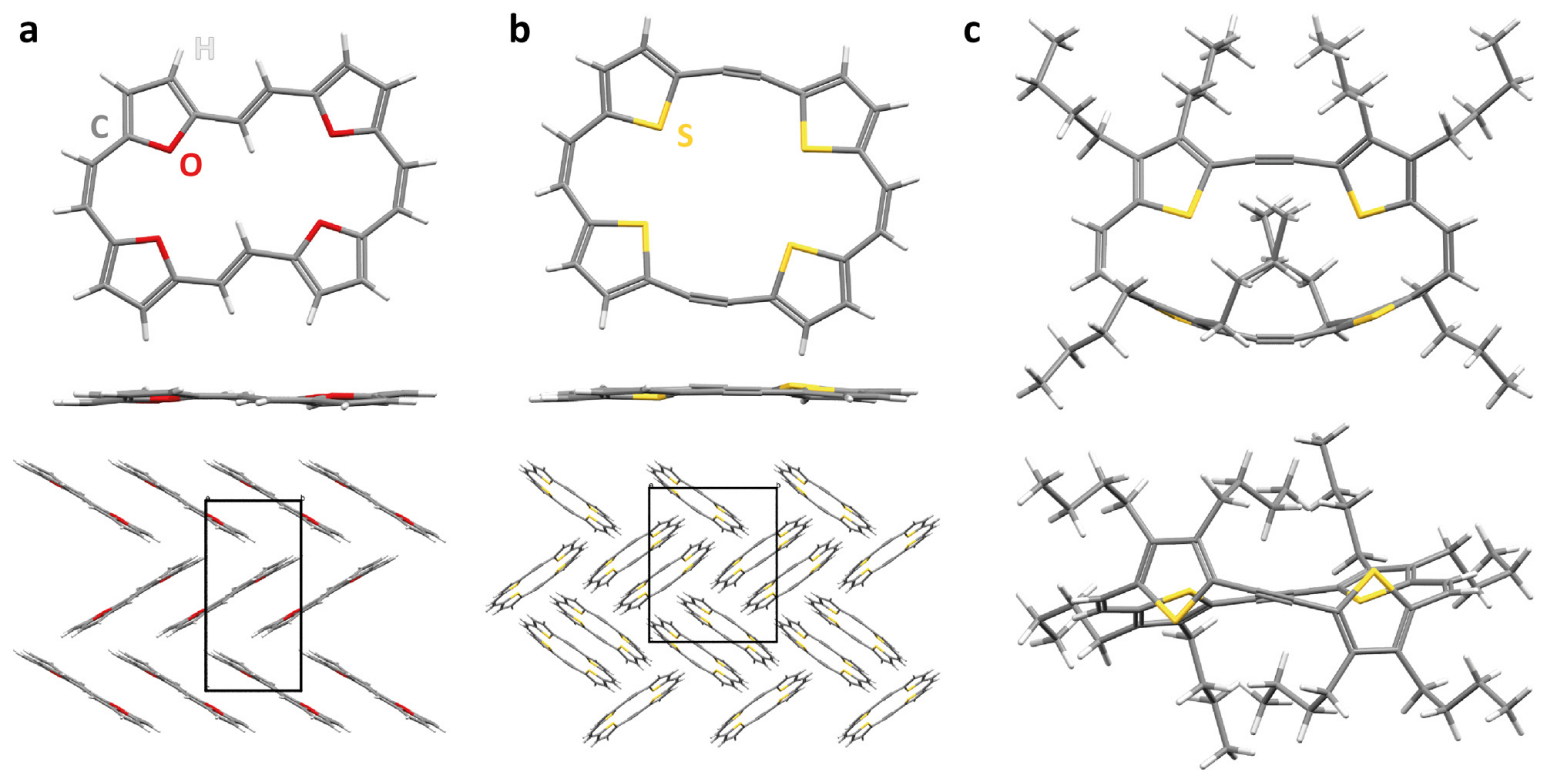
Crystal structures and packing of annulenes with concealed antiaromaticity. **a,b**, Crystal structure and packing of [24]annulene tetroxide in *cis,trans,cis,trans* configuration ( **a**) and 5,6,17,18-bisdehydrotetrathia[24]annulene ( **b**). The corresponding molecular structures are shown in Figure 3c. **c**, Crystal structure of 5,6,17,18-bisdehydrotetrathia[24]annulene with eight *n*-butyl chains attached to the thienylene units.

As an alternative to attaching bulky substituents, designing molecules with a formal 4 *n* π-electron system that fulfil the above requirements but – in contrast to Ni(II) norcorroles – are capable of conformational changes to further conceal the antiaromaticity (in addition to integrating fragments of the 4 *n* π-electron systems into locally aromatic subunits) may yield more stable molecules with Type III-CA. In solution, such molecules are expected to avoid coplanarity of the conformationally flexible aromatic subunits to conceal the antiaromaticity. In the solid state, however, full planarization of the molecules is expected to give rise to the energetically favourable intermolecular interactions that are characteristic for 4 *n* π-electron systems, potentially giving rise to stacked-ring aromaticity. Stable molecules with Type III-CA following this molecular design have been observed in the literature; the first macrocycle, [24]annulene tetroxide in *cis*, *trans*, *cis*, *trans* configuration ( Figure 3c), was reported to be virtually planar in the solid state ( Figure 4a) ^88^, indicating that it revealed its antiaromaticity for energetically favourable intermolecular interactions. The macrocycle is a configurational isomer of the all- *cis* macrocycle obtained when replacing the phenylene subunits of paracyclophanetetraene ( Figure 3a) with furanylene subunits. The furanylene subunits are less aromatic and less sterically demanding than phenylene subunits, which reduces the degree of concealment and allows for planarization in the solid state. In solution, however, [24]annulene tetroxide was reported to be highly flexible, enabling it to avoid planarity and to conceal its antiaromaticity to a large extent. The black-violet crystals of the macrocycle showed a melting point >300 °C and a staggered, chain-like face-to-face stacking ( Figure 4a bottom), resembling the stacking of methyl-substituted Ni(II) norcorrole ^83^. Besides Type III-CA, [24]annulene tetroxide also features Type I-CA according to ^1^H NMR measurements of its dication. It was reported that the “highly dynamic 24 π electron system of [the macrocycle] apparently loses its flexibility when it is oxidized”.

In contrast to [24]annulene tetroxide, 5,6,17,18-bisdehydro-tetrathia[24]annulene ( Figure 3c), another macrocycle that follows the proposed molecular design, was reported to crystallize in dimers with face-to-face interactions ( Figure 4b) ^89^, resembling the Ni(II) norcorrole dimers. The dark red needles of the compound showed a melting point of 242 °C. To the surprise of the authors reporting the structure, the macrocycle “has a planar X-ray structure with bent triple bonds and short S–S contacts” but “does not show antiaromatic (paratropic) properties” in solution, confirming that the antiaromaticity is effectively concealed in solution. Interestingly, the crystal structure of the corresponding macrocycle with eight sterically demanding *n*-butyl chains attached to the thienylene subunits has also been reported ( Figure 4c) ^90^. Here, the *n*-butyl chains are considered to hinder close interactions of the 4 *n* π-electron systems in the solid state, resulting in a non-planar, twisted conformation and in yellow crystals with a significantly lower melting point of 116 °C. This shows that macrocycles with concealed antiaromaticity that cannot interact closely with their neighbour(s) in the planar state or prefer other interactions will retain a non-planar conformation in the solid state and, hence, do not feature Type III-CA. However, oxidation of the macrocycle indicated that the compound does feature Type I-CA.

An expanded analogue of 5,6,17,18-bisdehydrotetrathia[24]annulene was also shown to crystallize in dimers ^91^, whereas a contracted analogue of [24]annulene tetroxide exhibited chain-like packing and Type I-CA ^92^. Recently, dimer interactions were also shown for a conformationally restricted phenylene-bridged hexaphyrin with concealed antiaromaticity, despite the presence of sterically demanding substituents ^93^.

## Practical utility of the concealed antiaromaticity conceptual framework

The concept of concealed antiaromaticity and the structural motifs and design strategies described in this article will enable the conscious, rational design of molecules that exhibit the desirable properties of antiaromatic molecules in redox reactions, upon photoexcitation, and in intermolecular interactions. Moreover, tailoring molecules and materials based on this conceptual framework can facilitate their preparation, processing, and application by avoiding or diminishing the undesirable low stability of antiaromatic compounds.

By considering the factors impacting the degree of concealment, molecules with different types of concealed antiaromaticity can be obtained, each type being ideal for different applications: (i) As shown for several molecules with Type I-CA, this type of concealed antiaromaticity is highly interesting for battery electrode materials, as the stability of the charged states is improved by the occurrence of global aromaticity, addressing a common issue of currently used organic battery electrode materials. At the same time, concealing the antiaromaticity in the neutral state ensures that the stability of the neutral state is not affected, enabling excellent reversibility of the redox reaction. It is envisioned that Type I-CA can also be an interesting feature for other applications that involve redox reactions, such as electrocatalysis and photoredox catalysis, where it may facilitate charge accumulation. For molecules with Type I-CA that undergo planarization upon reduction or oxidation, applications that can exploit this reversible conformational change are also promising. (ii) Similarly, molecules with Type II-CA are of interest for various applications, including singlet fission ^69^, as self-healing fluorophores ^94^, photoresponsive liquid crystals ^95, 96^, and molecular probes ^74, 97^. Applications in singlet fission exploit the low energy of the lowest triplet excited state relative to the energy of the lowest singlet excited state of molecules with Type II-CA, a property related to the Baird aromaticity. The relative energies of the two states can be tuned depending on the degree of concealment. Self-healing fluorophores can be obtained by covalently linking a fluorophore to a molecule with Type II-CA, which can act as a quencher of the otherwise long-lived, nonfluorescent triplet state that occurs if the fluorophore undergoes intersystem crossing. The process benefits from the Baird aromaticity of molecules with Type II-CA in the lowest triplet excited state, improving the performance of the fluorophore and avoiding the formation of reactive oxygen species. Photoresponsive liquid crystals and molecular probes both exploit the planarization of molecules with Type II-CA upon photoexcitation, requiring them to be conformationally flexible. (iii) Type III-CA is expected to be of particular interest for organic electronics and supramolecular architectures. Both applications utilize the close, energetically favourable intermolecular interactions of the π-electron systems of molecules with Type III-CA. In organic electronics, the effective intermolecular π-electron delocalization that can arise from these interactions is expected to enable materials with excellent intermolecular charge transport properties, potentially benefiting from stacked-ring aromaticity. In supramolecular chemistry, the energetically favourable intermolecular interactions can act as a driving force for self-assembly.

## Conclusion

The concept of concealed antiaromaticity helps resolve the argument about the terminology to be used for a class of molecules that feature both formal 4 *n* and [4 *n*+2] π-electron systems, without requiring a change of the definition of aromaticity and antiaromaticity. Not introducing a new type of (anti)aromaticity, the term describes that a molecule behaves like an antiaromatic molecule under certain conditions rather than describing the (anti)aromaticity of its neutral ground state. Thereby, the term is not limited to antiaromatic molecules but can also be suitable for locally aromatic or nonaromatic molecules if they show the described behaviour. They can be classified as molecules with strongly concealed antiaromaticity, as opposed to molecules with weakly concealed antiaromaticity. In order to avoid any confusion, it is recommended to use the phrase "molecules with concealed antiaromaticity" rather than "concealed antiaromatic molecules”.

In contrast to other terms that have been proposed, concealed antiaromaticity provides a more general framework for understanding and explaining the properties that arise from certain structural motifs, with a potential of being expanded to ‘concealed aromaticity’. It can also serve as a blueprint for others to develop and explore further, better molecules and materials for various research areas and applications.

## Ethics and consent

Ethical approval and consent were not required. 

## Data Availability

No data are associated with this article.
