# Supplementary material for: Concealed antiaromaticity
Source: Open Res Eur. 2025 Mar 18;5:70. [Version 1] doi: 10.12688/openreseurope.19690.1 (PMC12125571; doi:10.12688/openreseurope.19690.1)
Supplement: Peer review history

# Reviewer report: Reviewer response for version 1

**DOI:** 10.21956/openreseurope.21298.r52771

**Published:** 2025-06-09

**Authors:** Holger Helten

**Affiliations:** 1 Institut für Anorganische Chemie, Universität Würzburg, Würzburg, Germany

In this article the author proposes the introduction of “concealed antiaromaticity” as a terminology to describe the nature of molecules that could be antiaromatic regarding their 4n p-electron count contained within a closed loop but they are rather weakly antiaromatic or even non-aromatic, as their antiaromaticity is in some way diminished, however, under certain conditions they still do show characteristics typical of antiaromatic compounds. In my view this proposal is very appropriate and timely. It is a good approach to reconcile different, seemingly opposing viewpoints. Similar terms have been used before; however, they all seem to be less general or have a slightly different meaning than the concept introduced here. Notably, they have not been as clearly formulated as has been done in the present article. It is important to stress that it does not claim to be a proposal for a new definition of antiaromaticity, it merely represents a new terminology for molecules showing the properties mentioned. It should account for many compounds that may have been disregarded for a certain use where antiaromatic character could be exploited because they have been assessed weakly antiaromatic or non-aromatic, although diminishing – or concealing – their antiaromatic character may be particularly beneficial for the specific application. Indeed, we have just reported on a novel boraporphyrinoid to which the terminology proposed herein applies particularly well (refer to 1), and we have already taken it up for the description thereof. I generally support the concept proposed, and I believe that it will also be adopted by other researchers for the description of their own results – it may possibly even stimulate the design of new materials for specific applications.

 There are still some issues that I would like to point out. The following suggestions could help to further improve the present manuscript and/or to avoid misunderstandings:

 1) As mentioned before, there is a certain risk of misunderstanding of the proposed terminology as a new type or definition of antiaromaticity. This point is very clearly formulated in the Conclusion section. It would be advisable to formulate it in a similarly clear way early in the paper – perhaps at the end of the introduction.

 2) The other related concepts mentioned such as “antiaromaticity relief” should be cited and briefly discussed in the paper. The following is stated in this regard in the introduction: “Other terms that have been suggested in informal discussions, including attenuated antiaromaticity, pseudo-antiaromaticity, or concealed 4n p-electron cycles, are limited in scope and not suitable for the development of a more general framework.” It would be useful if the author could briefly explain what these terms have been proposed for and why they are considered unsuitable. Furthermore, there is no citation given. Have these terms never been defined in a written form? In that case, would it be useful to mention them here? Perhaps they should then be put in a footnote.

 3) The article contains four figures that each have three parts numbered as **a**, **b**, and **c**. If one skims quickly over the article, one could get the impression that there is an interconnection between them, especially between the three different structural motifs of concealed antiaromaticity and the three types of concealed antiaromaticity. I discussed this with a colleague who confirmed my view. For example, one could erroneously assume that it is claimed that molecules with the structural motif **a** conceal their antiaromaticity according to Type I-CA. Of course, it becomes clear that this is not the case when you read the article. However, this potential misinterpretation for the less careful reader can be avoided by using different types of numbering. Furthermore, in the text a different numbering, 1, 2, 3, is used rather than **a**, **b**, **c**. Actually, in the case of Figure 3, further numbering could be omitted, as the three types are already denoted as “Type I-CA” etc., so **a**, **b**, **c** and 1, 2, 3 are superfluous.

 4) There are some issues regarding the compilation of different types of structural motifs of concealed antiaromaticity presented in Figure 2. I agree that it is useful to make such a distinction; however, the classification provided is not comprehensive. For example, as mentioned before, heteroatoms are not considered. In our above-mentioned example, shared 4p-electron fragments are present, and the antiaromaticity is concealed by bridging sulfur atoms. Heteroaromaticity or sigma-aromaticity and other more “exotic” forms are not included into the classification provided. Furthermore, should a deviation from planarity also be regarded as a way to conceal antiaromaticity? I think it is not necessarily needed to provide a comprehensive classification, but it should be clearly stated that this is not the case. I suggest writing that the classifications cover “the most common motifs in organic chemistry” (or a similar formulation).

 Although I find it useful to include “internal connections” as a third classification in the figure, there are certainly ambiguities. For instance, biphenylene can be regarded as a twofold-internally connected [12]annulene or as a twofold-benzannulated cyclobutadiene (type **a**), respectively. There are several other possibilities for the second example as well. These ambiguities should be mentioned.

 5) A bit problematic is the point that it is often referred to different degrees of antiaromaticity concealment, but there is no criterion for quantification provided. Could perhaps the diradical character y_0 be utilized as suggested before?

 Page 4, top right: “… but the integration of 4 p-electrons is considered to have a more pronounced effect on the antiaromaticity than the integration of 2 p-electrons.” Could a reference be given for this claim or has this been noted here for the first time? Perhaps it could be explained how this conclusion has been drawn.

 Page 5: “The integration of fragments of the 4 *n *p-electron system into locally aromatic subunits is considered to lead to a tug-of-war between the local aromaticity of the subunits and the global antiaromaticity of the 4 *n *p-electron system, …” Could a reference be given for this claim? Can this be quantified? It has been noted that local aromaticity and global antiaromaticity may coexist in some cases.

 In the last section before the Conclusion, “Practical utility of the concealed antiaromaticity conceptual framework”, several examples of possible applications are provided. In the last passage of that section (page 8 bottom and page 9) there are no references given. I was wondering if some references are missing here, or have these suggested applications never been realized with this class of compounds yet? In that case, perhaps references where these applications have been realized in general (though with other compound classes) could be cited.

Where applicable, are recommendations and next steps explained clearly for others to follow? (Please consider whether others in the research community would be able to implement guidelines or recommendations and/or constructively engage in the debate)

Yes

Does the article adequately reference differing views and opinions?

Partly

Are all factual statements correct, and are statements and arguments made adequately supported by citations?

Partly

Is the rationale for the Open Letter provided in sufficient detail? (Please consider whether existing challenges in the field are outlined clearly and whether the purpose of the letter is explained)

Yes

Is the Open Letter written in accessible language? (Please consider whether all subject-specific terms, concepts and abbreviations are explained)

Yes

Reviewer Expertise:

Synthetic Inorganic / Organic Chemistry; Computational Chemistry; Macromolecular Chemistry

I confirm that I have read this submission and believe that I have an appropriate level of expertise to confirm that it is of an acceptable scientific standard, however I have significant reservations, as outlined above.

## References

[1] Buckel M, Klopf J, Schneider JS, et al.: A fully conjugated meso-boron-substituted porphyrinoid combining Lewis acidity with redox-switchable aromaticity. Nat. Commun. 2025; 16: 1775.10.1038/s41467-025-56892-wPMC1184009939971952

# Reviewer report: Reviewer response for version 1

**DOI:** 10.21956/openreseurope.21298.r52765

**Published:** 2025-05-30

**Authors:** Gokulnath Sabapathi

**Affiliations:** 1 Indian Institute of Science Education and Research Thiruvananthapuram, Vithura, Kerala, India

**Recommendation** **: *Accept with Minor Revisions***

 This work outlines a conceptual framework referred to as "concealed antiaromaticity," aiming to clarify discrepancies observed in the categorization of certain 4nπ-electron systems. Although these molecules are formally expected to exhibit antiaromatic behaviour based on electron-counting approaches, many deviate from this prediction. Their unexpected thermodynamic stability and the absence of typical antiaromatic features can be attributed to geometric and electronic modifications that hinder effective π-electron delocalization. The author proposes three forms of concealed antiaromaticity, each triggered by different conditions. **Type I-CA** appears during redox changes, **Type II-CA** under photoexcitation, and **Type III-CA** through intermolecular interactions.

 The article presents several strengths. It introduces the concept of "concealed antiaromaticity," offering a useful framework to explain π-systems that deviate from traditional aromaticity rules. The classification into three types of CA is logically structured and helps guide both molecular design and application. The paper also provides clear design principles based on structural motifs that influence antiaromatic behaviour. Moreover, the discussion effectively highlights potential uses in redox chemistry, molecular electronics, and photoresponsive materials. However, there are certain areas where the manuscript can be improved:

1.
  1. The paper does not define measurable parameters (e.g., NICS, diradical indices, HOMO–LUMO gaps) to assess the degree of antiaromaticity concealment, this can be included.
  2. The role of Baird aromaticity in **Type II-CA** is insufficiently addressed; a deeper theoretical and mechanistic discussion is expected before I arrive for a positive decision.
  3. It would be good if the authors can provide tabular summaries or comparative data to help evaluate and visualize examples more systematically.
  4. Some systems described as “concealed antiaromatic” may also fall under existing categories such as fluxional aromaticity or pseudoaromaticity. Discussing the overlap or differences with these existing terms would help the term “concealed antiaromaticity” with more distinctly within the reported examples.

Where applicable, are recommendations and next steps explained clearly for others to follow? (Please consider whether others in the research community would be able to implement guidelines or recommendations and/or constructively engage in the debate)

No

Does the article adequately reference differing views and opinions?

No

Are all factual statements correct, and are statements and arguments made adequately supported by citations?

Yes

Is the rationale for the Open Letter provided in sufficient detail? (Please consider whether existing challenges in the field are outlined clearly and whether the purpose of the letter is explained)

Yes

Is the Open Letter written in accessible language? (Please consider whether all subject-specific terms, concepts and abbreviations are explained)

Partly

Reviewer Expertise:

Pi conjugated planar antiaromatic and helically chiral macrocycles.

I confirm that I have read this submission and believe that I have an appropriate level of expertise to confirm that it is of an acceptable scientific standard.

# Reviewer report: Reviewer response for version 1

**DOI:** 10.21956/openreseurope.21298.r52770

**Published:** 2025-04-25

**Authors:** Roy Lavendomme

**Affiliations:** 1 Université libre de Bruxelles, Brussels, Belgium

In this article the author proposes the new terminology of “concealed antiaromaticity” to classify molecules that show “weakened” antiaromaticity to no (anti)aromaticity in their neutral ground state but still behave like antiaromatic compounds under certain conditions.

 This article is **not** about defining antiaromaticity or antiaromaticity criteria which is an entirely separate ordeal already undertaken by others in the literature attempting to complement the current IUPAC definitions.

 Overall, I appreciate the will to expand the compendium of scientific terminology to better categorize individual studies, thus facilitating literature research and discussion within the scientific community. In particular, when developing applications that make use of antiaromatic compounds properties, non aromatic compounds may be disregarded in early stages but, as clarified in this article, some may still display desired properties like antiaromatic compounds under specific conditions. Referring to such non aromatic compounds as “compounds with concealed antiaromaticity” may place them in the spotlight along antiaromatic compounds for application development.

 I am unsure if this concept of “concealed antiaromaticity” will find significant usage but it deserves to be proposed as, to the best of my knowledge, there is no equivalent existing concept (even if it partly overlaps with some terminology related to antiaromaticity).

 Some points addressed in the article are still unclear to me as listed below and should be clarified. If the concept is adopted by the community, it will likely be further refined in the long run.

1. It is made clear in the conclusion that this “concealed antiaromaticity” is not meant to define a new type of (anti)aromaticity nor a criterion of antiaromaticity. I would recommend to make such a clear disclaimer in the introduction to prevent readers from getting the wrong idea about what concept is presented therein. Indeed, reading the abstract, I first thought this concept was meant to describe weakened antiaromatic rings which then puzzled me when I saw examples of non aromatic compounds such as cyclooctatetraene.
2. The author regularly refers to different degrees of antiaromaticity concealment. Could the author clarify what the degree of concealment refers to and perhaps a criterion that could be used to categorize between weak and strong concealment? I presume (but could be wrong) that strongly concealed antiaromaticity refers to non aromatic molecules (e.g. bent cyclooctatetraene) whereas weakly concealed antiaromaticity refers to molecules containing weakly antiaromatic rings (e.g. tetraoxacirculene). In the introduction, the author presents the diradical character to define truly antiaromatic molecules but does not quantitatively refer to this criterion when addressing the degree of concealment.
3. I worry that using the word “antiaromaticity” for this concept may lead to confusion for non-experts when referring to molecules that do not meet criteria of antiaromaticity at any given point (e.g. non aromatic cyclooctatetraene). Perhaps the author could propose a solution to ensure that non experts would not wrongly assume that molecules with completely concealed antiaromaticity (i.e. non aromatic) are “antiaromatic”. I do believe that the recommendation made in the penultimate paragraph of the conclusion is insufficient. A solution could be to clearly state “non aromatic molecule with concealed antiaromaticity”, when applicable.
4. The difference between the structural motifs of concealed antiaromaticity “shared 2π-electron fragments” and “internal connections” appears to be arbitrary to me. Few examples:

- One could argue that the dibenzopentalene is a [16] annulene with 3 internal connections. Yet, it is presented in Fig 2c as being a cyclooctatetraene with 1 internal connection (= pentalene) and 2 shared 2π-electron fragments (with benzene rings).

 - Biphenylene is represented in Fig 2c as a cyclododecahexaene ([12] annulene) with two internal connections but it could be considered as a cyclobutadiene with 2 shared 2π-electron fragments (with benzene rings).

 - Pentalene (antiaromatic) could be viewed as a cyclooctatetraene (initially non aromatic) with 1 internal connection. This would mean that the structural motif of concealed antiaromaticity (i.e. internal connection) leads to an increase in antiaromaticity instead of decreasing it!

 I think that each structural motif for antiaromaticity concealment can be categorized in the first two types: shared 2π-electron fragments and shared 4π-electron fragments (perhaps with the addition of heteroatoms as a third category). The internal connections category is not useful, and I would personally remove it.

Where applicable, are recommendations and next steps explained clearly for others to follow? (Please consider whether others in the research community would be able to implement guidelines or recommendations and/or constructively engage in the debate)

Partly

Does the article adequately reference differing views and opinions?

Yes

Are all factual statements correct, and are statements and arguments made adequately supported by citations?

Yes

Is the rationale for the Open Letter provided in sufficient detail? (Please consider whether existing challenges in the field are outlined clearly and whether the purpose of the letter is explained)

Yes

Is the Open Letter written in accessible language? (Please consider whether all subject-specific terms, concepts and abbreviations are explained)

Yes

Reviewer Expertise:

Organic synthesis; Supramolecular chemistry; Reticular chemistry; Computational chemistry

I confirm that I have read this submission and believe that I have an appropriate level of expertise to confirm that it is of an acceptable scientific standard, however I have significant reservations, as outlined above.

# Reviewer report: Reviewer response for version 1

**DOI:** 10.21956/openreseurope.21298.r52314

**Published:** 2025-03-28

**Authors:** Miquel Solà

**Affiliations:** 1 University of Girona, Girona, Spain

In this paper, the author introduces the concept of concealed antiaromaticity. The work describes several antiaromatic systems that are stabilized in redox reactions, by photoexcitation, or by intermolecular interactions. To put my comments in context, let me first give the basis of my arguments:

1. I am not particularly in favor of introducing new forms of aromaticity except if they are strictly necessary (see Front. Chem. 5 (2017) 22).
2. I consider the best definition of aromaticity to be that provided by Chen et al. in 2005 (Chem. Rev. 105 (2005) 3842), who defined aromaticity as *“a manifestation of electron delocalization in closed circuits, either in two or in three dimensions. This results in energy lowering, often quite substantial, and a variety of unusual chemical and physical properties. These include a tendency toward bond length equalization, unusual reactivity, and characteristic* *spectroscopic features*.” Therefore, the two primary characteristics of aromaticity are aromatic stabilization and electronic delocalization, while ring currents are secondary features.
3. Aromatic stabilization is large in small rings and becomes residual in large rings (see J. Am. Chem. Soc. 143 (2021) 2403 & Chem. Sci., 16 (2025) 5613).

Having established the basis of my discussion, my comments are the following:

1. What is the difference between the term “concealed antiaromaticity” and “antiaromaticity relief” or “antiaromaticity alleviation” that has been used many times before the concept of “concealed antiaromaticity” was proposed (see for a few examples: Angew. Chem. Int. Ed. 60 (2021) 25082; Nat. Commun. 12 (2021) 5409; Chem. Sci. 15 (2024) 5225; J. Am. Chen. Soc. 142 (2020) 10942, etc.)? In all cases, the idea is that the antiaromaticity of a certain ring is reduced by redox reactions, photoexcitation, π-π interactions, or benzoannulation to explain the behavior of a certain compound. I do not see what the community gains by changing “antiaromaticity relief” to “concealed antiaromaticity.”
2. Let’s consider dibenzocyclooctatetraene in Figure 2a. I find the description of this molecule using the Glidewell-Lloyd rule (GLR) much more informative (see Theor. Chem. Acc., 135 (2016) 205) than the information I get from classifying it as a molecule with concealed antiaromaticity. With the GLR, I learn which is the best resonance structure to define this molecule, and, consequently, I learn which are the positions readier to react, for instance, as a dienophile in a Diels-Alder reaction. Same for tetraoxo[8]circulene. I find it much better to stay with the GLR rather than to change to the concealed antiaromaticity concept.
3. Ni(II) norcorrole is antiaromatic according to the magnetic criterion, but its stability comes from the local aromaticity of the dipyrril fragments (Sci. Rep. 9 (2019) 4852). In general, there is no tug-of-war between local aromaticity and global aromaticity as written in the manuscript. As to the aromatic stabilization energy, local aromaticity is more important than global aromaticity (see J. Am. Chem. Soc. 143 (2021) 2403 & Chem. Sci., 16 (2025) 5613), despite the magnetic criterion indicating the opposite in many situations.
4. I think it is wrong to classify paracyclophanetetraene (Figure 2b) as a molecule with concealed antiaromaticity. From the point of view of aromaticity, this molecule holds four π-sextets that provide about 120 kcal/mol of aromatic stabilization energy. The possible destabilization due to the antiaromaticity of the 24 π-electron circuit is minor (see J. Am. Chem. Soc. 143 (2021) 2403 & Chem. Sci., 16 (2025) 5613). Maybe one can classify paracyclophanetetraene as antiaromatic because of the existence of paratropic ring currents, but, if so (I do not know), then the classification is an artifact due to the use of ring currents as indicator of aromaticity. Same for cycloparaphenylenes.
5. Biphenylene is better described as an aromatic molecule with two π-sextets that become a 12π-electron Baird aromatic species in the lowest-lying triplet excited state (see J. Org. Chem., 82 (2017) 6327).
6. How can one distinguish between antiaromatic molecules and molecules with concealed antiaromaticity? All antiaromatic molecules are of types I-CA and/or II-CA and/or III-CA. If molecules with concealed antiaromaticity behave like antiaromatic molecules, then let’s call them antiaromatic.
7. I suggest the author mention that the aromaticity in π-π dimers is not related to “concealed antiaromaticity” but is caused by double-triplet [ ^1^(T _1_T _1_)] and intermolecular charge-transfer (CT) electron configurations mixing substantially in the ground state wavefunctions of the dimer (see Chem. Sci. 16 (2025) 1707).
8. I do not see how the concept of “concealed antiaromaticity” can improve our molecular designs. For instance, molecules with singlet fission properties have been designed with the current concepts about aromaticity without the need to invoke a new concept (see ref. 69). Same for the other molecular designs described.

Where applicable, are recommendations and next steps explained clearly for others to follow? (Please consider whether others in the research community would be able to implement guidelines or recommendations and/or constructively engage in the debate)

Yes

Does the article adequately reference differing views and opinions?

Partly

Are all factual statements correct, and are statements and arguments made adequately supported by citations?

No

Is the rationale for the Open Letter provided in sufficient detail? (Please consider whether existing challenges in the field are outlined clearly and whether the purpose of the letter is explained)

Yes

Is the Open Letter written in accessible language? (Please consider whether all subject-specific terms, concepts and abbreviations are explained)

Yes

Reviewer Expertise:

Theoretical and computational chemist

I confirm that I have read this submission and believe that I have an appropriate level of expertise to confirm that it is of an acceptable scientific standard, however I have significant reservations, as outlined above.
